# 2617. Antiviral usage for herpes simplex virus (HSV) detected in bronchoalveolar lavage fluid (BALF) by polymerase chain reaction (PCR) in hematologic malignancy (HM) and solid organ malignancy (SOM) patients with pulmonary infiltrates; impact of infectious disease consultation (IDC) on antiviral stewardship

**DOI:** 10.1093/ofid/ofad500.2230

**Published:** 2023-11-27

**Authors:** Cynthia Fu, Justine Abella Ross, Huiyan Ma, Bernard Tegtmeier, Jana Dickter, Deepa D Nanayakkara, Alfredo Puing, Avneet Kaur, Randy Taplitz, Sanjeet S Dadwal

**Affiliations:** City of Hope National Medical Center, Duarte, California; City of Hope National Medical Center, Duarte, California; City of Hope Comprehensive Cancer Center, DUARTE, California; City of Hope National Medical Center, Duarte, California; City of Hope National Medical Center, Duarte, California; COH, Duarte, California; COH, Duarte, California; City of Hope Comprehensive Cancer Center, DUARTE, California; City of Hope National Medical Center, Duarte, California; City of Hope National Medical Center, Duarte, California

## Abstract

**Background:**

HSV reactivation (HSVr) is frequent in HM and SOM patients while oropharyngeal/ mucocutaneous infections (OP-MCI) and especially HSV pneumonitis (HSVP) are uncommon. BALF-HSV-PCR can identify shedding or disease, however, may result in inappropriate high dose antiviral usage in non-HSVP situations. We report a cohort of patients with positive BALF-HSV-PCR, its impact on antiviral usage and role of IDC on antiviral use.

**Figure:**
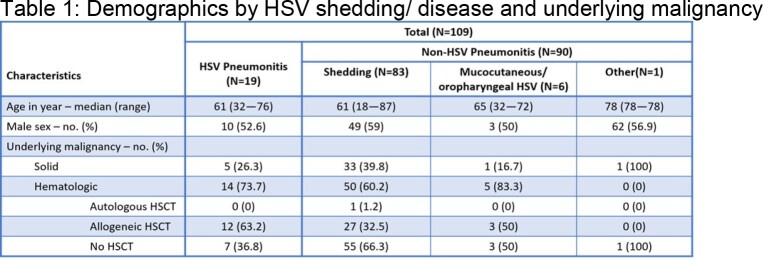

**Figure:**
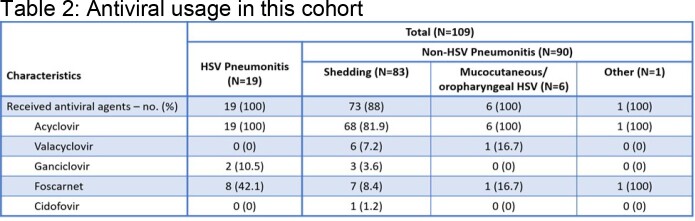

**Methods:**

Retrospective chart review of BALF-HSV-PCR positive patients who had IDC between 1/1/2018 and 8/31/2022. Exclusion criteria: No chest imaging (CT scan or chest X-ray imaging within ±7 days of positive test) and no malignancy. Data collection: demographics, laboratory and clinical findings, and antiviral medication dosing before and after each positive BALF-HSV-PCR preceding the IDC. Clinical diagnosis and treatment of HSVP versus non-HSVP was based on IDC. Kruskal-Wallis tests was used for continuous variables and Fisher’s exact test for categorical variables, and p-values reported were 2-sided at significance level of 0.05.

**Table 3.**
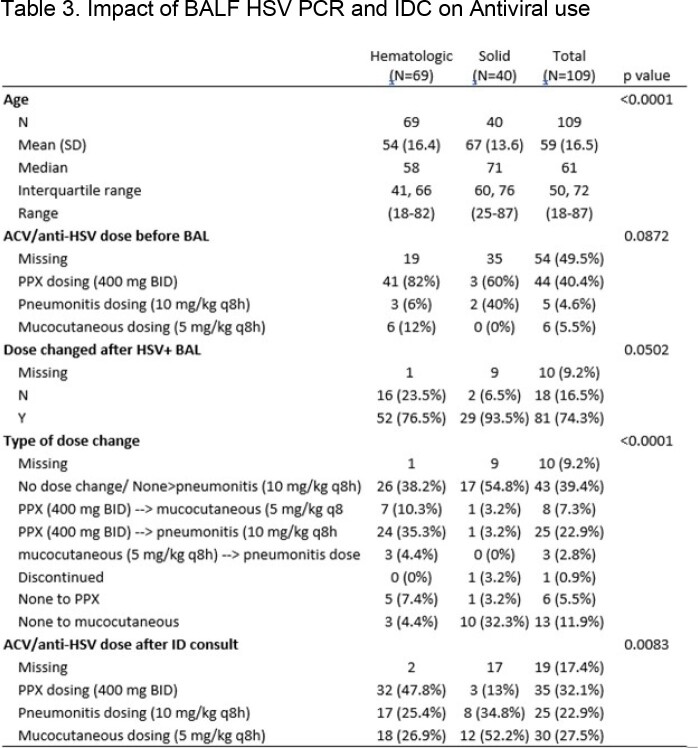
Impact of BALF HSV PCR and IDC on Antiviral use. PPX=prophylaxis, Y=yes, N=No; ACV=acyclovir

**Results:**

One-hundred-nine patients met inclusion criteria. Nineteen (17.4%) had HSVP, 90 (82.6%) non-HSVP. In non-HSVP; 83 (92%) had oropharyngeal shedding (HM >SOM), 6 (6%) OP-MCI and 1 (1%) immunotherapy related pneumonitis (IrP). Tables 1-3 provide details on demographics and baseline characteristics, antiviral use, and statistical analysis. HSV shedding/ disease (HSVP and OP-MCI) was more common in HM patients. All HSVP patients received high dose antiviral agents. Of ninety patients determined as non-HSVP, 42% were overtreated with antivirals with a positive test that preceded IDC. Positive PCR led to change in antiviral dose (p < 0.05) with highly significant impact on the dose of acyclovir used (prophylaxis vs. OP-MCI vs. HSPV doses) (p< 0.0001). IDC led to modification of HSVP dosing to either prophylaxis or complete discontinuation in 84.2% of patients (p=0.008).

**Conclusion:**

BALF-HSV-PCR positive test without HSVP led to overtreatment in both HM and SOM patients that is preventable by IDC, underscoring its value in antiviral stewardship. Diagnostic stewardship can further limit inappropriate use of antivirals.

**Disclosures:**

**Randy Taplitz, MD**, Karius: Advisor/Consultant|Merck: Advisor/Consultant|SNIPR biome: Advisor/Consultant **Sanjeet S. Dadwal, MD, FACP, FIDSA**, Allovir: Advisor/Consultant|Allovir: Grant/Research Support|Ansun Biopharma: Grant/Research Support|Aseptiscope, Inc: Stocks/Bonds|Astellas: Honoraria|Karius: Grant/Research Support|Matinas Biopharma: Stocks/Bonds|Merck: Advisor/Consultant|Merck: Grant/Research Support|Pfizer/Amplyx: Grant/Research Support|Takeda: Advisor/Consultant|Takeda: Honoraria|Viracor: Honoraria

